# Supplementary material for: The Systems Biology Research Tool: evolvable open-source software
Source: BMC Syst Biol. 2008 Jun 29;2:55. doi: 10.1186/1752-0509-2-55 (PMC2446383; doi:10.1186/1752-0509-2-55)
Supplement: Additional file 1 — SBRT Archive. An archive of the current version of the Systems Biology Research Tool. [file 1752-0509-2-55-S1.zip › sbrt-1.4.0/doc/developers_guide/api/sbrt/shell/io/RomoFile.html]

RomoFile


|  |  |  |
| --- | --- | --- |
| **Overview** \| **Package** \| **Class** \| **Use** \| **Tree** \| **Deprecated** \| **Index** \| **Help** | |  |
| **PREV CLASS**   **NEXT CLASS** | **FRAMES**    **NO FRAMES**     **All Classes** |  |
| SUMMARY: NESTED \| FIELD \| CONSTR \| METHOD | DETAIL: FIELD \| CONSTR \| METHOD |  |


---


## sbrt.shell.io Class RomoFile<K,V>

```
java.lang.Object
  sbrt.shell.io.AbstractMapOutputFile<K,V>
      sbrt.shell.io.RomoFile<K,V>
```

**Type Parameters:**: `K` - the key type.: `V` - the value type.

**All Implemented Interfaces:**: ResultsManager<java.util.Map<? extends K,? extends V>>, MapOutputFile<K,V>, MapTextFile<K,V>, MapTextOutputFile<K,V>, OutputFile<java.util.Map<? extends K,? extends V>>, SbrtFile

---

``` public class RomoFile<K,V> extends AbstractMapOutputFile<K,V> ```

This class is used to write maps to text files in a
row-oriented way.

**Author:**
:   This class was written and documented by
    Jeremiah Wright while in the Wagner lab.

---

**Constructor Summary**

`RomoFile(java.lang.String fileName,
FileFormat fileFormat,
MapFormat<K,V> vectorFormat)`
  
          Constructs a new row-oriented vector output file from the
provided arguments.

`RomoFile(java.lang.String fileName,
FileFormat fileFormat,
java.util.Set<? extends K> rowHeaderVariables,
MapFormat<K,V> mapFormat)`
  
          Constructs a new row-oriented map output file
from the provided arguments.

`RomoFile(java.lang.String fileName,
FileFormat fileFormat,
java.lang.String allRowHeaderVariables,
MapFormat<K,V> vectorFormat)`
  
          Constructs a new row-oriented vector output file
from the provided arguments.

`RomoFile(java.lang.String fileName,
MapFormat<K,V> vectorFormat)`
  
          Constructs a new plain text, row-oriented vector output
file from the provided arguments.


| **Method Summary** | |
| --- | --- |
| `void` | `addData(java.util.Map.Entry<? extends K,? extends V> variableValuePair)`             Writes the provided value to this file if the variable is contained in the set returned by `AbstractMapOutputFile.getFilterKeys()`. |
| `void` | `addData(java.util.Map<? extends K,? extends V> vector)`             Writes the appropriate values of the provided vector to this file. |

**Methods inherited from class sbrt.shell.io.AbstractMapOutputFile**

`breakPoint, close, getCanonicalName, getFilterKeys, getMapFormat, getName, getOriginalName, isClosed, writeLine`

**Methods inherited from class java.lang.Object**

`clone, equals, finalize, getClass, hashCode, notify, notifyAll, toString, wait, wait, wait`

**Methods inherited from interface sbrt.kernel.io.ResultsManager**

`addData`

**Constructor Detail**

### RomoFile

```
public RomoFile(java.lang.String fileName,
                FileFormat fileFormat,
                java.util.Set<? extends K> rowHeaderVariables,
                MapFormat<K,V> mapFormat)
```

:   Constructs a new row-oriented map output file
    from the provided arguments.

    **Parameters:**: `fileName` - the name of the file to create.: `fileFormat` - the format of the file to create.: `rowHeaderVariables` - the variables whose values will be written to this file. If `null`, all values from every added vector will be written to this file.: `mapFormat` - the vector format. **Throws:**: `java.lang.NullPointerException` - if any argument except `rowHeaderVariables` is `null`.

---


### RomoFile

```
public RomoFile(java.lang.String fileName,
                FileFormat fileFormat,
                java.lang.String allRowHeaderVariables,
                MapFormat<K,V> vectorFormat)
```

:   Constructs a new row-oriented vector output file
    from the provided arguments.

    **Parameters:**: `fileName` - the name of the file to create.: `fileFormat` - the format of the file to create.: `allRowHeaderVariables` - a string representation of the variables whose values will be written to this file. This string is parsed by the provided vector format.: `vectorFormat` - the vector format. **Throws:**: `java.lang.NullPointerException` - if any argument is `null`.

---


### RomoFile

```
public RomoFile(java.lang.String fileName,
                FileFormat fileFormat,
                MapFormat<K,V> vectorFormat)
```

:   Constructs a new row-oriented vector output file from the
    provided arguments. The value of each variable from every added
    vector will be written to this file.

    **Parameters:**: `fileName` - the name of the file to create.: `fileFormat` - the format of the file to create.: `vectorFormat` - the vector format. **Throws:**: `java.lang.NullPointerException` - if any argument is `null`.

---


### RomoFile

```
public RomoFile(java.lang.String fileName,
                MapFormat<K,V> vectorFormat)
```

:   Constructs a new plain text, row-oriented vector output
    file from the provided arguments. The value of each variable
    from every added vector will be written to this file.

    **Parameters:**: `fileName` - the name of the file to create.: `vectorFormat` - the vector format. **Throws:**: `java.lang.NullPointerException` - if either argument is `null`.


**Method Detail**

### addData

```
public void addData(java.util.Map.Entry<? extends K,? extends V> variableValuePair)
```

:   Writes the provided value to this file if the variable is
    contained in the set returned by `AbstractMapOutputFile.getFilterKeys()`.

    :   **Parameters:**: `variableValuePair` - the variable-value pair. **Throws:**: `java.lang.IllegalArgumentException` - if the provided vector does not contain all the variables in the set returned by `AbstractMapOutputFile.getFilterKeys()`.: `java.lang.IllegalStateException` - if `close()` has already been called.

---


### addData

```
public void addData(java.util.Map<? extends K,? extends V> vector)
```

:   Writes the appropriate values of the provided vector
    to this file. The appropriate values are those of the
    variables in the set returned by `AbstractMapOutputFile.getFilterKeys()`.

    :   **Parameters:**: `vector` - the vector to be written to this file. **Throws:**: `java.lang.IllegalArgumentException` - if the provided vector does not contain all the variables in the set returned by `AbstractMapOutputFile.getFilterKeys()`.: `java.lang.IllegalStateException` - if `close()` has already been called.


---


|  |  |  |
| --- | --- | --- |
| **Overview** \| **Package** \| **Class** \| **Use** \| **Tree** \| **Deprecated** \| **Index** \| **Help** | |  |
| **PREV CLASS**   **NEXT CLASS** | **FRAMES**    **NO FRAMES**     **All Classes** |  |
| SUMMARY: NESTED \| FIELD \| CONSTR \| METHOD | DETAIL: FIELD \| CONSTR \| METHOD |  |


---
